# The Performance of FDA-Approved PET Imaging Agents in the Detection of Prostate Cancer

**DOI:** 10.3390/biomedicines10102533

**Published:** 2022-10-10

**Authors:** Mei Li, Roman Zelchan, Anna Orlova

**Affiliations:** 1 Department of Medicinal Chemistry, Uppsala University, 751 23 Uppsala, Sweden; 2 Liaoning Medical Device Test Institute, Shenyang 110171, China; 3 Department of Nuclear Medicine, Cancer Research Institute, Tomsk National Research Medical Center, Russian Academy of Sciences, 5 Kooperativny St., 634009 Tomsk, Russia; 4 Research Centrum for Oncotheranostics, Research School of Chemistry and Applied Biomedical Sciences, Tomsk Polytechnic University, 634050 Tomsk, Russia; 5 Science for Life Laboratory, Uppsala University, 752 37 Uppsala, Sweden

**Keywords:** prostate cancer, positron emission tomography, imaging agent

## Abstract

Positron emission tomography (PET) incorporated with X-ray computed tomography (PET/CT) or magnetic resonance imaging (PET/MRI) is increasingly being used as a diagnostic tool for prostate cancer (PCa). In this review, we describe and evaluate the clinical performance of some Food and Drug Administration (FDA)-approved agents used for visualizing PCa: [^18^F]FDG, [^11^C]choline, [^18^F]FACBC, [^68^Ga]Ga-PSMA-11, [^18^F]DCFPyL, and [^18^F]-NaF. We carried out a comprehensive literature search based on articles published from 1 January 2010 to 1 March 2022. We selected English language articles associated with the discovery, preclinical study, clinical study, and diagnostic performance of the imaging agents for the evaluation. Prostate-specific membrane antigen (PSMA)-targeted imaging agents demonstrated superior diagnostic performance in both primary and recurrent PCa, compared with [^11^C]choline and [^18^F]FACBC, both of which target dividing cells and are used especially in patients with low prostate-specific antigen (PSA) values. When compared to [^18^F]-NaF (which is suitable for the detection of bone metastases), PSMA-targeted agents were also capable of detecting lesions in the lymph nodes, soft tissues, and bone. However, a limitation of PSMA-targeted imaging was the heterogeneity of PSMA expression in PCa, and consequently, a combination of two PET tracers was proposed to overcome this obstacle. The preliminary studies indicated that the use of PSMA-targeted scanning is more cost efficient than conventional imaging modalities for high-risk PCa patients. Furthering the development of imaging agents that target PCa-associated receptors and molecules could improve PET-based diagnosis of PCa.

## 1. Introduction

Every year, 1.3–1.4 million men are diagnosed with prostate cancer (PCa) and more than 0.3 million die from it [1]. PCa prevalence is influenced by age, racial factors, and regional factors. One in eight American men will be diagnosed with PCa during their lifetimes and this proportion significantly increases to six in ten for men who are older than 65. The incidence rate increases from 155 per 100,000 in men aged 55–59 to 751 per 100,000 in men aged between 75–79, and PCa-associated morbidity also increases with age [2]. The statistics show that Asians have the lowest PCa morbidity. In Europe, 22% of all the cancers diagnosed in 2018 were PCa. African-Americans have the highest proportion of incidences in the US (according to the American Cancer Society’s estimation in 2021) [3]. Japanese men who live in the US have more PCa than those who live in Japan, due to their diet pattern [4]. 

These high incidence rates have attracted intensive investigations into the diagnosis and treatment of PCa. The disease can be stratified into indolent and aggressive types, of which aggressive PCa correlates with poor prognosis and short overall survival (OS). There are no suitable biomarkers available in clinics to differentiate the aggressive form of disease from the indolent one. In the early stages, the localized disease can be cured; however, a large proportion of cases will metastasize to the lymph nodes, bones, and other organs. Some patients will develop castration-resistant PCa, which will be incurable. Common treatment strategies used for PCa include (but are not limited to) the following: radical prostatectomy (RP), androgen deprivation therapy (ADT), chemotherapy, and radiotherapy (RT). A precise diagnosis of the cancer site and disease stage will influence the treatment decision and therapeutic outcome. Hence, it is of vital importance to develop accurate diagnostic methods in order to select and manage the treatment strategy. 

Prostate-specific antigen (PSA) blood testing is widely used in the screening, active surveillance and treatment-response monitoring of PCa. However, the effectiveness of PSA testing is still under hot debate. PSA is a protein that is found in prostate cells and is normally confined to the prostate gland. Elevated PSA levels can be found in the blood when PCa, prostatitis, benign prostate proliferation, and/or various other conditions (such as old age or riding a bicycle) are present. Therefore, PSA is only an indicator of PCa and not an ultimate diagnostic biomarker. Statistics show that most men without PCa have PSA levels under 4 ng/mL and that levels of 4–10 ng/mL and over 10 ng/mL suggest a 25% and 50% chance, respectively of having PCa. However, 15% of patients with PCa also have PSA levels below 4 ng/mL [5]. Therefore, the PSA value of 4 ng/mL, which is typically used to determine PCa, is not actually a reliable threshold value. Further tests are needed for men with increased PSA values, which may include digital rectal examination, imaging, and biopsy. 

The conventional imaging modalities used for PCa diagnosis include transrectal ultrasound (TRUS), magnetic resonance imaging (MRI), and X-ray computed tomography (CT). The first imaging technique for PCa localization is TRUS, which can be used for T-staging (localized tumor stage), according to the European Association of Urology’s (EAU’s) guidelines [6,7]. A TRUS-guided biopsy is a standard and cost-effective method for the diagnosis of PCa and has been widely used in the past three decades. The Gleason score (GSC) is used to describe the disease’s progression based on biopsy material. Patients are then sorted into risk levels according to their PSA values and GSCs, and the chosen treatment is based on these risk levels [8]. For patients with a low risk, active surveillance is considered to be the gold standard, while for patients with an intermediate risk, the suitable treatment includes RP and RT. Patients in the high-risk group are subjected to a combination of active treatment and systemic therapy. However, a prostate biopsy is invasive and associated with the potential risk of infection. Furthermore, cancer can be underestimated or missed in biopsy samples; in only 28.7% of cases did the GSCs obtained from an initial TRUS-guided biopsy match those obtained from RP specimens [9]. Hence, diagnostic approaches involving imaging modalities (which can provide visible information about the cancer site) have been introduced to supplement the information obtained by biopsy. 

Driven by the requirement for precise staging of the tumor, MRI was employed for MRI-directed and MRI-guided biopsies. The MRI-directed TRUS-guided biopsies showed higher detection rates (25%) than TRUS-guided biopsies without initial MRI-imaging (9%); moreover, no false negatives were observed for MRI-directed TRUS-guided biopsies [10]. The MRI-guided biopsy is performed using real-time MRI guidance, and the detection rate for this method has been reported to be 40–60% [11]. However, despite the sampling advantages during these MRI-guided/directed biopsies, the staging of PCa by CT and MRI depends on the morphology of the malignant tissue, and only 2.5% of histologically proven lymph node metastases (LNM) can be detected by CT [12]. Due to the low sensitivity and specificity of these conventional imaging modalities, novel diagnostic approaches that can identify and classify PCa with high accuracy are under extensive study. 

Unlike conventional imaging modalities (which generally give anatomical and morphological information about the disease), radioisotope-based imaging, such as positron emission tomography (PET) and single-photon emission computed tomography (SPECT), can provide information at the molecular level. Radioisotope-based imaging approaches provide information that includes changes in blood flow and oxygen supply, increases in energy consumption, an increased need for components required for cell proliferation, and increases in specific proteins expressed in or on cells. However, the limitation of these methods is that radioisotope-based imaging will not provide anatomical information about the disease. Therefore, radioisotope-based imaging modalities are often coupled with CT or MRI to increase their sensitivity and specificity. 

Currently, radionuclide imaging plays an important role in both PCa diagnosis (primary staging of the tumor and restaging after recurrence) and PCa prognosis (monitoring treatment response and predicting the outcome). Numerous tracers have been produced and evaluated for PCa, which can be divided into three subcategories: (i) imaging agents that are used to detect cell division activity (e.g., choline, acetate, glucose, and amino acids); (ii) radiolabeled molecules that are used to target cancer-specific membrane proteins or receptors, e.g., ligands that bind to androgen receptors (ARs), prostate-specific membrane antigen (PSMA), or gastrin-releasing peptide receptors (GRPRs); and (iii) compounds that specifically bind to bone metastases [13]. Table 1 lists the radioisotope-based agents that are used for the visualization of PCa with PET and SPECT. With these tracers, information regarding PCa characterization and localization, as well as treatment response, can be obtained. 

However, each of the tracers has a particular indication and limitation. To provide more precise information about the disease, imaging needs to be tailored to the specific phases of the disease in a patient-specific manner [14]. The PET imaging modality provides higher resolution than SPECT, due to the detection of antiparallel photons emitted by the annihilation of the positron. Commonly used PET imaging agents are labeled with C-11, F-18, and Ga-68. These isotopes, with a short half-life, provide a lower radiation dose for the patient. In addition, the incorporation of C-11 or F-18 into the imaging molecule will not influence its biological properties, such as the binding affinity to the target. In the past decade, PET imaging technology developed rapidly in many fields, including the PCa field. Some of the tracers have been approved by the Food and Drug Administration (FDA) and European Medical Agency (EMA), while some are still under development. The focus of this article is to evaluate different PET imaging agents approved for clinical use, in terms of specificity, sensitivity, detection rate, and suitable clinical use.

## 2. Materials and Methods

A comprehensive search strategy was carried out with a screening window set from 2010 to March 2022 using https://pubmed.ncbi.nlm.nih.gov/ from 1 January 2010 to 1 March 2022. For some of the early PET tracers, the search date started with the first publication of that tracer. The searching strategy and the keywords were as follows: Prostate cancer + PET + Tracer name. From all studies published in English, we selected the articles that were associated with the discovery, preclinical study, clinical study, and evaluation of the agent’s diagnostic performance. Imaging agents will be described according the accumulation mechanism in PCa, as mentioned above.

## 3. Results

### 3.1. Imaging Agents Targeting the Cell Division and Proliferation

**Figure 1:**
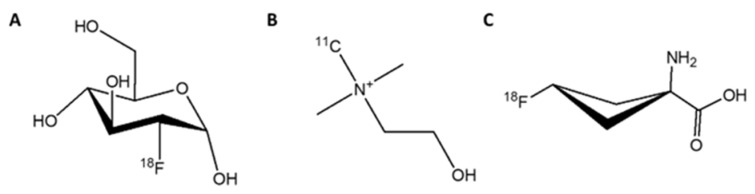
PET tracers approved by the FDA for imaging of PCa that accumulate in cancer cells due to cell division. (**A**) [^18^F]FDG, (**B**) [^11^C]choline; (**C**) [^18^F]fluciclovine ([^18^F]FACBC).

Cancer is characterized by the unregulated cell proliferation that can spread from the primary site to other tissues or organs. The cell division and proliferation expend more energy, amino acids, and cell membrane components. Therefore, the molecules involved in cell division and proliferation can be used as tracers for molecular imaging when labeled with a radioisotope. These are glucose, choline, acetate, and amino acids. The tracers in this category that have been approved by the FDA include ^18^F-FDG, ^11^C-choline, and ^18^F-fluciclovine (Figure 1). 

#### 3.1.1. [^18^F]FDG

**Figure 2:**
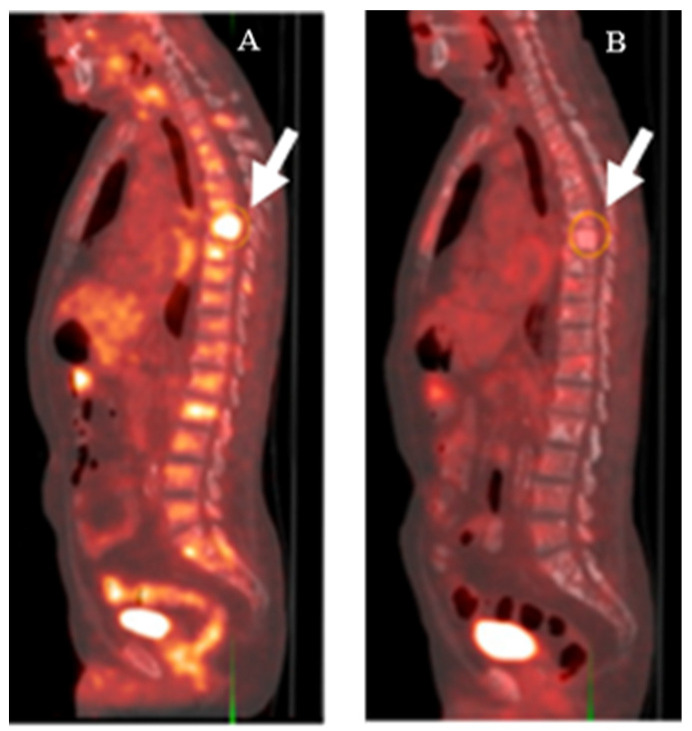
[^18^F]FDG-PET imaging of a patient with CRPC before (**A**) and after (**B**) treatment with docetaxel. Metabolic activity in thoracic spine lesions (white arrows) decreased after treatment. This research was originally published in the Journal of Nuclear Medicine (JNM) [17].

Fluorodeoxyglucose labeled with flourine-18 ([^18^F]FDG) is an analog of glucose, which can be used to trace cells and tissues with high glucose consumption (Figure 1A). It is approved by the FDA for PET imaging in oncology, cardiology, and neurology [16]. [^18^F]FDG accumulates in the cells via glucose transporters located on the cell membrane (Figure 2). This tracer is easily accessible and is widely used to detect many tumors, such as lymphoma, breast cancer, lung cancer, and others. However, PCa often grows slowly and its glucose transporter expression is lower than it is in other types of malignant cells, which leads to low tumor-to-background ratios and decreases the detection rate of PCa when it is imaged with [^18^F]FDG. Moreover, the tracer cannot differentiate between malignant PCa and benign prostatitis (because the inflammation process also increases the glucose uptake) which may result in false-positive images and a subsequent decrease in specificity [14]. Therefore, the utility of [^18^F]FDG in the initial diagnosis and staging of PCa is rather limited.

Despite the limited use of [^18^F]FDG in the diagnosis of PCa, some studies have been performed to investigate the potential use of [^18^F]FDG as an auxiliary indicator in the field of PCa. The more recent investigations of [^18^F]FDG as an imaging agent for PCa focused on its use as a prognostic agent. Jadvar et al. stated that in patients with castration-resistant PCa, the sum of their maximum standardized uptake values (SUV_max_) for all detectable lesions (a parameter derived after subtracting an SUV baseline value for normal hepatic tissue) negatively correlated with their OS [18]. Similarly, it was also shown that the time to hormone therapy failure (THTF) significantly decreased with increases in these summed SUV_max_ values for metabolically active lesions [19]. The role of [^18^F]FDG imaging in the treatment management of PCa has also been studied. Hillner et al. reported that in 35% of PCa patients, after imaging, their management was changed from treatment to nontreatment, or vice versa. However, that was still lower than for other cancers, such as small cell lung cancer and myeloma [20]. Recently, it was reported that despite only moderate detection rates of 16.7% for [^18^F]FDG PET/CT scans used on PCa patients with negative [^68^Ga]Ga-PSMA-11 PET/CT scans, the patients with positive [^18^F]FDG scans possessed higher PSA levels and GSCs compared to patients with negative scans [21]. These data suggest that [^18^F]FDG-imaging of PCa could be used as an auxiliary diagnostic tool in patients with high PSA values and GSCs, but who had produced negative PSMA-based PET/CT results.

#### 3.1.2. [^11^C]Choline

**Figure 3:**
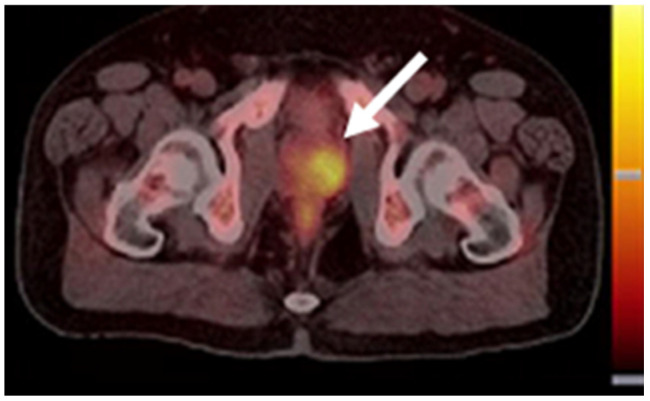
[^11^C]Choline PET/CT image of a patient with an adenocarcinoma of the prostate (white arrow). This research was originally published in the Journal of Nuclear Medicine (JNP) [29].

Choline is an essential nutrient involved in the synthesis of phosphatidylcholine, a vital component of the cell membrane (Figure 3). As tumor cells require more cell membrane material than normal tissues, due to their accelerated proliferation, the consumption of choline is increased. [^11^C]Choline is synthesized by substituting one of the carbon atoms in the methyl group of choline with the radioisotope carbon-11 (Figure 1B) and was first employed as a PET tracer for PCa in 1998 [22]. [^11^C]Choline imaging demonstrated better sensitivity (52%) and similar specificity (99%) than MRI (18.5% and 99%, respectively) in the nodal staging of PCa [23]. The effectiveness of [^11^C]choline in PCa detection has been confirmed in small groups of patients. Scattoni et al. showed that [^11^C]choline PET/CT imaging possessed a high detection accuracy of 77% in patients with lymph node metastases [24]. In a prospective study of patients with lymph node dissection as the salvage therapy after primary treatment by RP failed, the correct prediction value was 53% for [^11^C]choline PET/CT scans [25]. The detection rates were ~80% in two retrospective studies of patients with biochemical recurrence (BCR) or patients with a suspicious local relapse of PCa after a RP [26,27]. These studies demonstrated that [^11^C]choline PET/CT imaging may be useful in restaging patients with PCa relapse. In 2013, [^11^C]choline was approved by the FDA for use in PET scans, in order to detect sites of recurrent PCa [28]. 

However, the detection rate for PCa recurrence dropped to 55% when [^11^C]choline was applied to a large cohort of PCa patients (3203 patients) [30]. The detection rate for [^11^C]choline was found to be associated with PSA values. A detection rate of 73% was found for patients with PSA levels above 2.0 ng/mL [26], but this rate dropped to only 27% for patients with PSA levels below 1.16 ng/mL [30] and further decreased for patients with even lower PSA levels [31]. It is still unclear to what extent [^11^C]choline PET/CT imaging can be useful in the diagnosis of BCR PCa, especially if biochemical relapse for PCa patients after an RP is defined as patients having PSA levels higher than 0.2 ng/mL. However, despite the low detection rate, a negative [^11^C]choline scan can predict a longer OS after a prostatectomy and ADT [31,32]. [^11^C]Choline PET/CT may be useful in monitoring patients after primary curative therapy and in the detection of BCR, but unsuitable for the primary diagnosis of PCa [32].

#### 3.1.3. [^18^F]Fluciclovine (FACBC)

**Figure 4:**
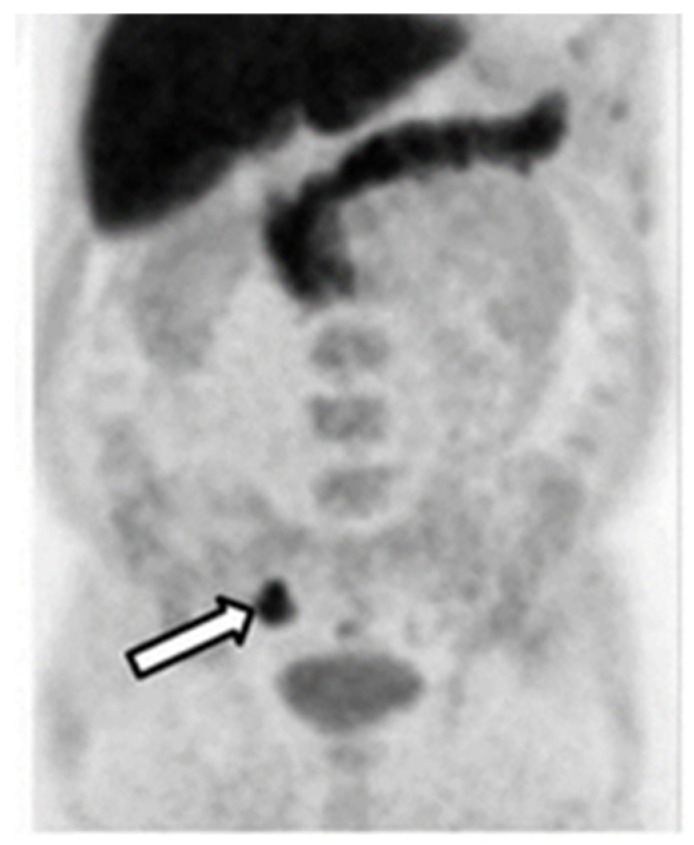
A [^18^F]fluciclovine PET/CT image of PCa (white arrow). This research was originally published in the Journal of Nuclear Medicine (JNM) [36].

A radiolabeled analog of the essential amino acid leucine, [^18^F]FACBC (Figure 1C) was developed and first used to image a patient with a brain tumor [33]. [^18^F]FACBC accumulates in tumor cells, due to their upregulation of amino acid transporters, and can be used as an imaging agent for several types of tumors, including PCa (Figure 4). In a prospective study that compared the accuracy of [^18^F]FACBC and [^11^C]choline in the detection of PCa relapse, the detection rate was 32% for [^11^C]choline versus 38% for [^18^F]FACBC [34]. Moreover, the production of [^18^F]FACBC is easier than [^11^C]choline, and the longer half-life of fluorine-18 is more favorable in clinical use. In 2016, [^18^F]FACBC was approved by the FDA for the detection of suspected recurrent PCa [35].

The diagnostic performance of [^18^F]FACBC was widely assessed after the FDA’s approval. The reported overall detection rate for [^18^F]FACBC ranged from 40% to 80%, depending on the size of patient cohorts, and like [^11^C]choline, its detection rate positively correlated with patients’ PSA levels(see Table 2 [37,38,39,40,41,42,43]). In a study investigating BCR patients with very low PSA levels (≤0.3 ng/mL), the detection rates were 43.8% for patients with PSA level below 0.1 ng/mL, increasing up to 65.2% for patients with PSA between 0.2 and 0.3 ng/mL [44]. In patients with BCR, an [^18^F]FACBC PET/CT scan led to mainly major changes in clinical management for 40% of patients [45].

The diagnostic performance of [^18^F]FACBC was compared to that of [^11^C]choline PET/CT for the primary staging of PCa. The respective sensitivity, specificity, and accuracy were 50%, 70%, and 65% for [^11^C]choline and 50%, 81%, and 74% for [^18^F]FACBC [45]. Similar results were reported for preoperative lymph node staging in PCa patients: [^18^F]FACBC showed patient-based and region-based sensitivity/specificity values of 40%/100% and 30%/100%, respectively [46].

The high specificity of [^18^F]FACBC PET imaging could be used to follow treatment after an RP. However, its low sensitivity in preoperative PCa patients means that not all lymph node metastases (LNM) could be detected even in patients with a positive [^18^F]FACBC PET scan; therefore, the extended pelvic lymph node dissection cannot be waived.

The detection rate when using [^18^F]FACBC correlates with the tumor size: detection rates of 23.7% and 83.3% for metastases with the respective diameters of 3 mm and 9 mm have been reported [47]. Taking into account that about 90% of LNM are smaller than 5 mm [48], the suitability of [^18^F]FACBC for staging is limited.

It can be concluded that imaging agents that target tissue with high energy, amino acid, and membrane-component consumption do not demonstrate high specificity for PCa. 

### 3.2. Imaging Agents Targeting Cancer-Specific Membrane Proteins or Receptors

Several PCa-targeted PET tracers have emerged that bind specifically to cancer-specific membrane proteins or receptors: PSMA, gastrin-releasing peptide receptor (GRPR), AR, urokinase plasminogen activator (uPA), prostate stem cell antigen (PSCA), the six-transmembrane epithelial antigen of the prostate (STEAP1–4), and CD46 have been investigated extensively within the last few decades. Among these, the FDA-approved agents that image PSMA expression have revealed the most promising diagnostic potential. 

PSMA is a cell-surface glycoprotein that is highly expressed on the PCa cell’s membrane and presents in various tumors, e.g., breast cancer, bladder cancer, glioblastomas, gastric carcinoma, and colorectal carcinoma [49,50]. It is also endogenously expressed in healthy organs, such as the kidneys, the small intestine, and the central and peripheral nervous systems. However, its expression on malignant cells is much higher than on the normal cells [50]. PSMA is negatively regulated by androgen and positively correlated with GSCs [49], thus making PSMA a promising theranostic target in PCa. 

**Figure 5:**
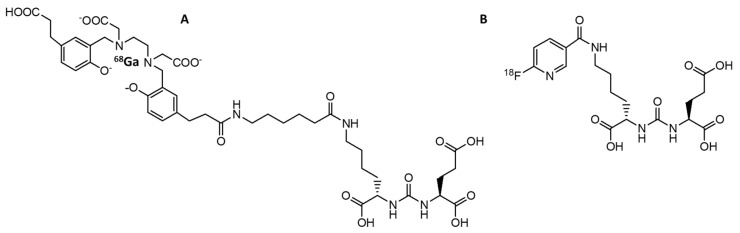
PET tracers approved by the FDA for PSMA-targeted imaging of PCa. (**A**) [^68^Ga]Ga-PSMA-11; (**B**) [^18^F]DCFPyL.

**Figure 6:**
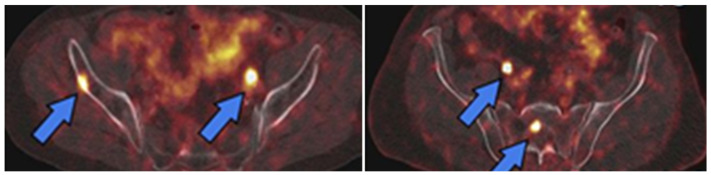
PET images of PSMA expression in PCa (blue arrows) using [^68^Ga]Ga-PSMA-11 (left image) and [^18^F]DCFPyL (right image). This research was originally published in the Journal of Nuclear Medicine (JNM) [61].

The first PSMA-targeted imaging agent, capromab pendetide (with the brand name ProstaScint^®^), was a monoclonal antibody derived from the murine one. In 1996, [^111^In]-capromab pendetide was approved by the FDA as a SPECT imaging agent for PCa and it was used to evaluate patients with a high risk of metastasis or a high clinical suspicion of recurrence. However, the sensitivity of [^111^In]-capromab pendetide in the diagnosis of PCa was only 17% [51]. The reason for the low sensitivity was that the binding site of [^111^In]-capromab pendetide is located on the intracellular domain of PSMA; hence, a positive imaging result using this agent could also be due to the presence of dead/dying cells in the cancer tissue [52,53]. Therefore, the application of [^111^In]-capromab pendetide is limited. Subsequently, four monoclonal antibodies (J591, 415, 533, and E99) targeting PSMA were identified by Liu et al. [53]. These new antibodies only bound to viable cells, indicating that their binding sites were in the extracellular domain of PSMA. The J591 antibody was labeled with zirconium-89 [54] for PET imaging of PCa, indium-111 for a SPECT imaging [55], and lutetium-177 for the treatment of metastatic castration-resistant PCa [56]. However, the intrinsic long blood circulation times of full-length antibodies which resulted in low-contrast images, as well as the appearance of smaller PSMA-targeted pseudopeptides (Figure 5), changed the direction of development of PSMA-imaging agents toward small molecules that specifically target PSMA [57,58]. Based on evidence regarding safety and efficacy, two PSMA-targeted pseudopeptides were recently approved by the FDA for PCa imaging (Figure 6): [^68^Ga]Ga-PSMA-11 [59] and [^18^F]DCFPyL [60]. 

#### 3.2.1. [^68^Ga]GaPSMA-11 

The pseudopeptide [^68^Ga]Ga-PSMA-11 (Figure 2A) consists of two parts, the PSMA binding motif and the HBED-CC-chelator for labeling with gallium-68 [62]. [^68^Ga]Ga-PSMA-11 possesses a rapid blood and organ clearance, tumor-specific accumulation, and high tumor-to-background ratios. The kidneys, salivary glands, lacrimal glands, liver, and intestine possess high-to-moderate tracer uptake as healthy organs [59].

The lesion detection rate was 60% for patients with PSA < 2.2 ng/mL, which was comparable with the detection rates for [^11^C]choline and [^18^F]FACBC [26,37,38,40,41,42]. However, for patients with PSA > 2.2 ng/mL, [^68^Ga]Ga-PSMA-11 performed superiorly, with a detection rate of 100% [59]. In December 2020, [^68^Ga]Ga-PSMA-11 was approved by the FDA for patients with suspicious PCa metastasis and PCa recurrence after curative-intent therapy [63]. 

Current investigations are focused on the diagnostic accuracy of PCa before an RP, because the initial detection of PCa is very important for disease management. The initially reported sensitivity for [^68^Ga]Ga-PSMA-11 for primary diagnosis in intermediate- or high-risk PCa ranged from 40% to 93%, while the specificity was consistently high (between 88% and 99%) [64,65,66,67,68,69,70,71,72]. However, a recently published meta-analysis of seven studies reported a pooled sensitivity of 97%, but a somewhat lower specificity of 66%, for the initial detection of PCa using [^68^Ga]Ga-PSMA-11 [73]. Therefore, more data is needed to reliably evaluate the effectiveness of [^68^Ga]Ga-PSMA-11 in the primary diagnosis of PCa.

PET/CT imaging using [^68^Ga]Ga-PSMA-11 has also demonstrated its utility for treatment management. In patients who underwent an RP, ADT, or RT, the detection rate was 84–100% for all clinical scenarios (except for patients with PSA levels < 0.2 ng/mL after an RP, where the detection rate was only 8%). The treatment change in the full cohort was 57% after imaging with [^68^Ga]Ga-PSMA-11 [74]. In another study, [^68^Ga]Ga-PSMA-11 could predict the treatment outcome for RP better than conventional imaging, including [^11^C]choline PET/CT, contrast-enhanced CT, bone scan, or abdominal MRI [75].

Several retrospective and prospective studies identified a positive correlation between detection rates for [68Ga]Ga-PSMA-11 imaging and PSA values in patients with BCR (see Table 3). In the studies included in our review, the overall detection rates showed a broad range from 34.4% to 94%. In studies with median PSA values of 0.32–0.65 ng/mL, the overall detection rates were 35–50% [75,76,77]. This was lower than in studies that had median PSA values of 0.99–10.65 ng/mL, wherein the detection rates were 71–94% [72,73,74,75,76,77]. A meta-analysis identified a similar trend, with pooled detection rates of 63% for PSA < 2 ng/mL and 94% for PSA > 2 ng/mL [78]. The variation in detection rates in the above-mentioned studies could be a reflection of the heterogeneity status of patients’ PSMA expression. 

Due to the wide range of reported data, the factors associated with the detection rate of [^68^Ga]Ga-PSMA-11 were explored. Parameters such as PSA value, ADT, patient’s age, GSC, and injected peptide dose were investigated, and the correlation of these parameters with positive PET scans were analyzed in a large-cohort retrospective study. The results demonstrated that only log PSA values and ADT were associated with positive PET results [76]. However, a correlation between ADT and the probability of a positive PET scan with [^68^Ga]Ga-PSMA-11 should be interpreted with caution, because ADT is likely to be used in patients with advanced disease. The impact of the imaging time points (1 h vs. 3 h after injection) on the detection rate was also investigated. At 3 h post-injection, more lesions were visualized than at 1 h: 147 lesions in 68 patients vs. 134 lesions in 57 patients, respectively [77]. More than 60% of the PCa lesions displayed higher SUV_max_ and better contrast at 3 h than at 1 h post-injection. However, approximately 14% of the lesions revealed lower SUV_max_ and worse contrast at 3 h post-injection (but they were still clearly visible). This phenomenon was not investigated further in the study.

A head-to-head comparison of [^68^Ga]Ga-PSMA-11 with [^18^F]FACBC (which accumulates in dividing cells) revealed a somewhat better overall detection rate for [^68^Ga]Ga-PSMA-11 PET/CT—82.8% vs. 79.3%, respectively [79]. However, in patients with local recurrence, [^18^F]FACBC identified more lesions (37.9%) than [^68^Ga]Ga-PSMA-11 (27.6%). This could be due to the accumulation of activity in the urinary bladder. The obstacle of high activity in the bladder’s content could be overcome by hydration and injection of diuretics (e.g., furosemide) before scanning [77]. The detection of pelvic lymph node recurrence, extra pelvic lymph node metastases, and bone metastases using [^68^Ga]Ga-PSMA-11 was superior to that of [^18^F]FACBC. In another study, [^68^Ga]Ga-PSMA-11 detected five more positive patients than [^18^F]FACBC detected; furthermore, all the patients with positive [^18^F]FACBC PET/CT scans were also identified by the [^68^Ga]Ga-PSMA-11 scan [80].

#### 3.2.2. [^18^F]DCFPyL 

The PSMA-targeted imaging agent labeled with fluorine-18, [^18^F]DCFPyL (PYLARIFY^®^) (Figure 2B) has the same PSMA binding motif as for [^68^Ga]Ga-PSMA-11. However, fluorine-18 has a higher positron abundance and lower positron energy compared with gallium-68; hence, [^18^F]DCFPyL demonstrates better spatial resolution compared with that of [^68^Ga]Ga-PSMA-11 [88]. The longer half-life and wide availability of fluorine-18 is additionally beneficial for the implementation of this tracer. The first-in-man study showed its safety and efficacy in the application for PCa diagnosis, without any severe adverse events detected within 30 days post-injection [89]. 

In a phase II/III clinical trial involving high risk PCa patients (OSPREY), [^18^F]DCFPyL detected pelvic lymph node metastases with moderate sensitivity (40.3%) but high specificity (97.9%) [90].

Imaging with [^18^F]DCFPyL was further evaluated in a clinical trial known as CONDOR. Patients with BCR, median PSA levels of 0.8 ng/mL, and an uninformative prior imaging result (by one of the conventional imaging modalities such as CT, MRI, whole-body bone scintigraphy, [^11^C]choline, or [^18^F]FACBC PET) were included. The treatment plan was changed in 50% of the patients after a positive PSMA scan and in 13.6% of the patients who obtained negative PSMA scans [91]. Accordingly [^18^F]DCFPyL demonstrated its superiority over other conventional imaging modalities, as well as its reliability for the detection of the BCR of PCa. As with other mentioned tracers, the detection rate for [^18^F]DCFPyL correlated with patients’ PSA levels and increased from 36% for PSA < 0.5 ng/mL to 97% for PSA > 5 ng/mL [91], which was similar to previously published results for [^68^Ga]Ga-PSMA-11 [92]. In a performance evaluation of [^18^F]DCFPyL PET/CT, in patients with recurrent PCa and low PSA levels, the detection rates were 63.6% (<0.2 ng/mL), 58.7% (0.2–0.49 ng/mL), 62.8% (0.5–0.99 ng/mL), 82.1% (1–1.99 ng/mL), and 91.7% (>2 ng/mL) [5], which were higher than for primary staging [90]. Recently, a meta-analysis reviewing the diagnostic performance of [^18^F]DCFPyL in the early detection of BCR showed a pooled detection rate of 81% [93]. The author further stratified the detection rate by PSA values; the pooled detection rate was 88.8% for PSA values > 0.5 ng/mL and 47.2% for the PSA value < 0.5 ng/mL. [^18^F]DCFPyL PET possesses higher detection rates, especially in groups with low PSA values (<0.5 ng/mL), when compared with other foregoing PET-imaging agents for PCa. In May 2021, [^18^F]DCFPyL was approved by the FDA for patients with suspected PCa metastases who are candidates for initial definitive therapy, and for patients with suspected recurrence after curable treatment showing increasing serum PSA levels [94].

Despite its short period in clinical use, [^18^F]DCFPyL has been evaluated in several studies. In patients with PSA values > 3.6 ng/mL, the diagnostic performance of [^18^F]DCFPyL in the primary staging of PCa demonstrated a 51% detection rate for lymph node metastases (LNMs), a 56% detection rate for distant metastases, and a consequential influence on several patients’ treatment [95]. Moreover, approximately half of the LNMs that were positively detected by [^18^F]DCFPyL did not show signs of lymph node enlargement on CT scans; therefore, [^18^F]DCFPyL demonstrated better accuracy in the primary staging of PCa than did CT. [^18^F]DCFPyL PET demonstrated superiority in comparison with conventional imaging for the staging of and treatment decision in PCa patients with recurrent or metastatic disease [90]. Of the patients staged as M0 by the conventional imaging modality, 58% were upgraded to M1 after [^18^F]DCFPyL PET/CT imaging, and 16% of patients who were initially staged as M1 showed an increased stage after a [^18^F]DCFPyL scan. A small cohort study (in patients with high-risk PCa) investigated whether [^18^F]DCFPyL PET/MRI allowed distinguishing between benign prostate hyperplasia and malignant tumors [96]. The time–activity curves from dynamic [^18^F]DCFPyL PET showed a continuous increase in activity uptake in tumors and a persistent decrease in activity uptake in benign prostate hyperplasia and normal tissues. 

A comparison between the two PSMA-targeted radiopharmaceuticals ([^18^F]DCFPyL and [^68^Ga]Ga-PSMA-11) in the detection of BCR demonstrated that a [^18^F]DCFPyL PET scan following a [^68^Ga]Ga-PSMA-11 PET scan identified at least nine more additional lesions, due to a higher tumor-to-background contrast [88].

The detection of bone metastases with [^18^F]DCFPyL in comparison to [^18^F]NaF has also been investigated. There were no statistically significant differences in the detection of osseous metastatic sites; however, [^18^F]DCFPyL PET could provide additional information in soft tissues [97]. The findings in this study suggested a potential role for [^18^F]FDCPyL in the detection of bone metastases in PCa patients. To confirm whether the PSMA-based tracer actually performed more accurately than radiolabeled sodium fluoride in the detection of bone metastases, more clinical data are needed.

### 3.3. Imaging Agents Targeting Bone Lesions

With the progression of PCa, lymphogenous (affecting regional lymph nodes) and hematogenous (affecting internal organs and bones) metastases appear. Most commonly, the PCa metastases are detected in the osseous. Approximately 80% to 90% of patients who die from PCa have developed bone metastases [98]. The early detection of bone metastases would be helpful in tailoring patient’s treatments and, therefore, improving their survival and their quality of life. In addition to approving the easily accessible and inexpensive ^99m^Tc-labeled SPECT tracers for bone scans (which have been widely used to detect any bone involvement in PCa), the FDA has also approved a PET tracer for bone imaging, [^18^F]-NaF. 

#### [^18^F]-NaF

**Figure 7:**
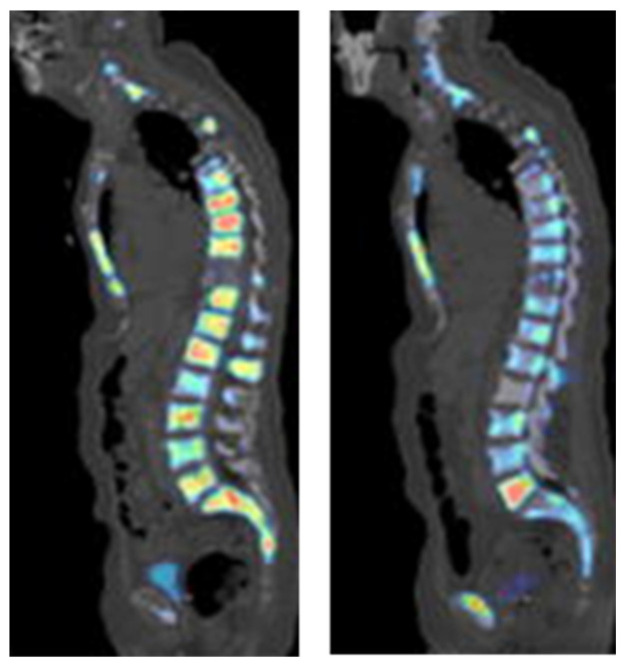
[^18^F]Fluoride PET imaging of a PCa patient before (left image) and after (right image) treatment with dasatinib. This research was originally published in the Journal of Nuclear Medicine (JNM) [99].

[^18^F]-NaF was first applied in diagnosing osteogenic activity four decades ago. The mechanism of action for [^18^F]-NaF in the detection of bone lesions, including metastases, is based on the chemisorption of [^18^F]F^−^ into hydroxyapatite, a bone component. Because of limited development of PET cameras at the time, this agent was not widely used and, therefore, other technologies, such as bone scintigraphy and SPECT were employed [98]. However, the specificity of SPECT was low and its acquisition times were long. In the last ten years, with the development of PET scanner technology, [^18^F]-NaF, was approved by the FDA for the indication of altered osteogenesis. It has been extensively applied for bone lesion detection in PCa (Figure 7). The performance of this imaging agent has been investigated in the following scenarios: for the detection of bone metastases in PCa patients, for the monitoring of treatment response, and for other aspects of utility, such as in combination with other imaging agents, for the detection of PCa.

In order to evaluate the diagnostic performance of [^18^F]-NaF in bone metastases of PCa, Park et al. prospectively studied men with or without bone metastases that had been classified based on a [^99m^Tc]Tc-MDP bone scan (TcBS). In the patient cohort with a negative TcBS result, approximately 50% showed evidence of bone metastases when scanned using [^18^F]-NaF [100], and these results were similar to those published by Kim et al. [101]. In Kim et al.’s study, patients with PCa also had both [^18^F]-NaF and TcBS scans. Regarding the lesion-based analysis, there were more bone lesions identified by [^18^F]-NaF than were identified by TcBS in 70% of the patients. Regarding the patient-based analysis, the positive detection rate was 80% for the [^18^F]-NaF scan and 47% for the TcBS scan. The detection of PCa bone metastases using [^18^F]-NaF PET/CT was shown to be superior to the detection using TcBS. This trend has also been seen in various other studies [102,103].

The pooled sensitivity and specificity for [^18^F]-NaF was 98% and 90%, respectively, which were higher than the sensitivity and specificity for both ^99m^Tc-bone scintigraphy and ^99m^Tc-SPECT or SPECT/CT [103]. When [^18^F]-NaF was compared with ^68^Ga[Ga]-PSMA-11 PET/CT in the detection of osseous metastases, the detection rate was about 30% higher for [^18^F]-NaF [104]. However, another study demonstrated that in PCa patients who had inconclusive results from [^18^F]-NaF PET/CT scans, an additional [^68^Ga]Ga-PSMA-11 PET/CT scan was able to precisely diagnose and change the patients’ management with an accuracy of 96% [105]. It has also been shown that [^18^F]-NaF can be useful in monitoring the treatment–response to chemotherapy with dasatinib in castration-resistant prostate cancer (CRPC). In that study, baseline scans were compared, with a scan taken 12 weeks after treatment, and the SUV_max_ had significantly decreased in metastatic bone lesions but not in normal bones [99]. Recently, it was also demonstrated that the combined use of [^18^F]-NaF and [^18^F]FACBC could detect soft tissue metastases and bone lesions simultaneously. The sensitivity and specificity of the combination increased, compared with either imaging agent used separately [106].

Finally, a comparison of the three PET tracers, [^18^F]DCFPyL, [^18^F]-NaF, and [^18^F]FDG, in the diagnosis of metastatic PCa was performed by Fourquet et al. [107]. [^18^F]-NaF (with a bone lesion detection rate of 90.5%) was superior to [^18^F]DCFPyL (which detected 46.4% of the bone lesion) and ^18^F-FDG (which detected only 28.0% of all lesions). The imaging concordance between [^18^F]-NaF and [^18^F]DCFPyL was only 50%, but that was still higher than the 22.2% between [^18^F]DCFPyL and [^18^F]FDG. This overall discordance of imaging with different tracers demonstrates the degree of heterogeneity in PCa metastases. These observations further support the combined use of tracers that could increase detection rates and provide more information to clinicians in making decisions regarding patient-centered therapy.

## 4. Discussion

The earlier FDA-approved PET tracers for PCa were only recommended for BCR until the emergence of PSMA-targeted tracers, which have been approved for the indication of suspected PCa metastasis before curative surgery, as well as suspected PCa recurrence. The higher detection rates for PSMA-targeted imaging agents versus other previously used PET-imaging agents was demonstrated by both prospective and retrospective clinical trials. The diagnosis of PCa patients with PSA values under 1 ng/mL remains the obstacle for the majority of imaging agents. However, the PSMA-targeted imaging agents have shown better detection rates in this subgroup and significantly higher detection rates overall. Based on preliminary clinical data, several meta-analyses have summarized the diagnostic performance of [^11^C]choline, [^18^F]FACBA, [^68^Ga]Ga-PSMA-11, and [^18^F]DCFPyL. According to the literature study in this review, the PSMA-targeted imaging agents reveal better detection accuracy with higher pooled sensitivity, pooled specificity, and pooled detection rates; see Table 4. 

Among the clinically approved PSMA-targeted agents, [^18^F]DCFPyL identified more lesions and revealed better tumor-to-background ratios than [^68^Ga]Ga-PSMA-11 [88]. However, the existence of Ge/Ga generators enables extending the reach of PSMA-targeted scanning to facilities that do not possess expensive cyclotrons. 

Further improvement in diagnostic accuracy for radiolabeled PCa imaging agents could be expected with wilder use of next generation cameras, i.e., PET cameras integrated with MRI anatomical option and digital PET/CT (dPET/CT) cameras equipped with SiMP (silicon photomultiplier) detectors. It was reported that [^68^Ga]Ga-PSMA-11 PET/MRI was particularly more efficient than PET/CT in the detection of local recurrences in the prostate bed, due to the better imaging of soft tissue with MRI. However, the overall detection rate was just slightly better for PET/MRI (67.9%) than for PET/CT (64.2%) [113]. dPET cameras provide better spatial and time resolution and better sensitivity than traditional analog PET cameras. dPET allows not only discrimination between malignant and benign tissues in the prostate gland, but also optimizes the imaging time window [114]. [^68^Ga]Ga-PSMA-11 dPET/CT demonstrated a higher detection rate than traditional PET/CT in similar cohorts of PCa patients and performed better in patients with PSA below 2 ng/mL [115]. In high-risk patients with early CRPC who did not have lesions detected by PET/CT and/or bone scan, [^18^F]FACBC dPET/CT identified PCa lesions in 87.5% of this cohort [116].

The comparison between PSMA-targeted imaging agents and [^18^F]-NaF for the detection of bone metastases was inconclusive [97,104,107]. In order to confirm whether PSMA-targeted imaging agents could actually replace [^18^F]-NaF, more clinical data would be needed. However, the tracers targeting PSMA could detect additional hematogenous metastases in the liver, lungs, brain, and bone marrow, which could be beneficial for patients without bone involvement.

PCa is highly heterogeneous; therefore, the precise detection of PCa with PET/CT using only one imaging agent is still a challenge in clinical use. PSMA is not universally expressed by all PCa cells. It was reported that 10% of patients with metastatic CRPC will produce a false negative result when using PSMA-based PET/CT for detection [117]. Further investigations should be conducted to overcome this obstacle.

In an attempt to solve this heterogeneity problem, the combined use of two imaging agents were employed to increase detection rates. For patients with negative or equivocal [^18^F]fluorocholine PET/CT scans and low PSA values (<0.2 ng/mL), an additional [^68^Ga]Ga-PSMA-11 PET/CT scan produced a positive result in 41% of the patients [118]. Furthermore, overall detection rates were increased when using dual-tracer ([^68^Ga]Ga-PSMA-11 and [^18^F]FDG) PET scans in castrated patients, compared with a single scan using only one of the agents [119]. As already mentioned, [^18^F]-NaF PET/CT scans can be combined with PSMA-targeted agents to visualize soft-tissue lesions [105]. In such combinations, the limitations owing to the absence of a universal PET-imaging agent for PCa could be addressed.

**Figure 8:**
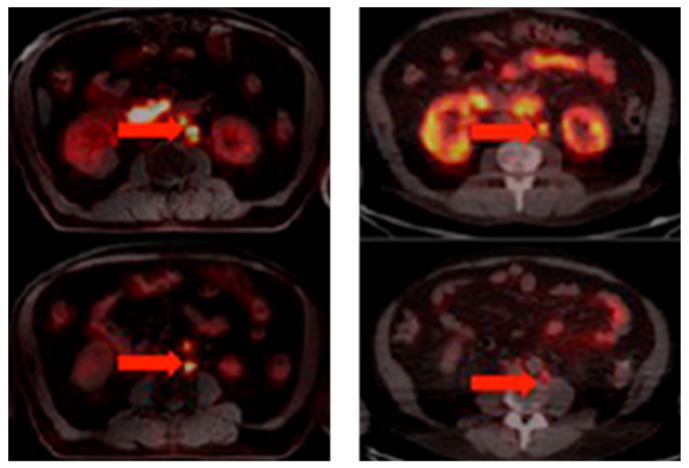
[^68^Ga]Ga-RM2 (left) vs. [^68^Ga]Ga-PSMA-11 (right) PET/CT images of PCa lesions (red arrows) in a patient with LNM. This research was originally published in the Journal of Nuclear Medicine (JNM) [122].

The development of PCa-specific imaging agents evolved rapidly. One particular group of tracers with the potential for PCa detection is that of the bombesin receptor antagonists, which target the GRPR. Despite the lower overall proportion of PCa exhibiting GRPR expression versus PSMA, the overexpression of GRPR is already found in primary tumors, while PSMA expression increases with PCa progression [120,121]. The bombesin receptor antagonist [^68^Ga]Ga-RM2 revealed PET/CT scan results that were similar to those using [^68^Ga]Ga-PSMA-11 in a small group of PCa patients (Figure 8) [122]. Moreover, compared with [^68^Ga]Ga-PSMA-11 (which accumulated in the salivary glands, lacrimal glands, and small intestine), the physiological uptake of [^68^Ga]Ga-RM2 was limited to the pancreas. [^68^Ga]Ga-RM2 could also provide more information about the retroperitoneal lymph nodes than the PSMA-targeted tracer. GRPR-targeted imaging agents may be a supplementary tracer for PSMA-negative patients. 

Another promising target in the PCa field is CD46, which possesses the ability to downregulate the innate immune response and is overexpressed in metastatic CRPC. In a recent investigation, the anti-CD46 (fully human full-length IgG1) antibody YS5, labeled with the radioisotope zirconium-89, demonstrated superior tumor-to-background ratios than [^68^Ga]Ga-PSMA-11 in murine models [123]. A phase I clinical trial aimed at studying the imaging performance of [^89^Zr]Zr-DFO-YS5 in metastatic CRPC patients is currently recruiting patients (NCT05245006). Once complete, this study should determine the optimal time point for imaging, as well as the optimal dose for this tracer. The study will also investigate the tracer’s sensitivity in the detection of metastatic lesions. In addition, an antibody–drug conjugate (ADC) based on YS5 is undergoing phase I clinical trials (NCT03575819). 

The good diagnostic performance of PSMA-targeted tracers and the high tumor expression of PSMA in advanced PCa have facilitated the investigation of radiotherapeutics that target PSMA, such as [^177^Lu]Lu-PSMA617 and [^225^Ac]Ac-PSMA617 [124,125,126]. However, responses to RT are generally reflective of the heterogeneity of PSMA expression in PCa tumors. The influence of PSMA expression on RT responses has been confirmed by Current et al. [117]. Therefore, PSMA-targeted PET tracers could be used in the stratification of patients for RT that targets PSMA, and in monitoring the treatment response.

Finally, pseudopeptide heterodimers targeting two different receptors/antigens specific to PCa could help in overcoming the problem with PCa heterogeneity. Relatively small pseudopeptides, combining targeting moieties for PSMA and GRPR, were investigated in preclinical murine models with PCa, and promising data were reported in the literature [127,128,129,130]. However, future investigations could focus on the discovery of PCa-specific biomarkers to discriminate between indolent and aggressive PCa, as well as on the development of the “ideal” imaging agent that could target PCa using a target that is uniformly expressed in PCa.

A large proportion of PCa patients (classified as high-risk in primary staging using conventional imaging) will develop BCR after an RP or RT. This rapid recurrence of the disease indicates that the disease burden or its aggressiveness has been underestimated. PSMA-targeted imaging agents have shown superior sensitivity and specificity in both the primary staging of PCa and the detection of PCa recurrence, compared with conventional imaging modalities. However, the extent to which high-risk PCa patients would benefit from PSMA-targeted imaging as a first-line imaging modality is still less evident. A cost-comparison analysis carried out in Australia showed that for patients with high-risk PCa (who will be treated with RP or RT), the total cost of both a CT and bone scan will be AUD 1412, while a [^68^Ga]Ga-PSMA-11 PET scan will cost AUD1203 [131]; using [^18^F]DCFPyL will even further decrease the cost for each PET scan.

## 5. Conclusions

Currently, there are six PET-imaging agents for PCa diagnosis: [^18^F]FDG, [^11^C]choline, [^18^F]FACBC, [^68^Ga]Ga-PSMA-11, [^18^F]DCFPyL, and [^18^F]-NaF. Each tracer has its role in the field of PCa. However, the PSMA-targeted radiopharmaceuticals demonstrate superiority over the other approved imaging agents in their diagnostic performance for primary staging, detection of recurrence at an early stage, and identification of bone lesions at an advanced stage. Even though [^18^F]-NaF detects more bone lesions, the imaging agents targeting PSMA are the most versatile tracers that are now available. The literature reported several novel applications of PET-imaging agents in PCa: one was combining a PSMA-targeted imaging agent with a second imaging agent for the more precise staging of PCa; another was using a pair of radioisotopes for both the imaging and therapy (theranostic) with PSMA-targeted molecules. A preliminary cost-effectiveness study for [^68^Ga]Ga-PSMA-11 PET clearly demonstrated the superiority (in both diagnostic accuracy and price) of PSMA-targeted imaging agents over conventional imaging agents as a first-line imaging modality. With the development of more imaging agents that specifically target PCa cells, richer clinical evidence supporting the benefits of new-generation imaging agents, and the broader availability of PET/CT, the utility of PET imaging in the field of PCa will definitely increase. 

## Figures and Tables

**Table 1:** The radioisotope-based imaging agents for PCa.

Target	Mechanism of Accumulation in Tumors	Compounds
Choline	Choline is used to synthesize phosphatidylcholine, a cell membrane component. Overexpression of choline kinase has been observed in human PCa	^11^C- and ^18^F-choline
Acetate	Acetate is the substrate of lipid and cholesterol synthesis. In PCa cells, the increased cellular uptake of acetate was found	^11^C-acetate, ^18^F-acetate
Glucose	Glucose is a ubiquitous fuel in the human body, particularly for hypermetabolic cells, and increased expressions of transmembrane glucose transporters and hexokinase have been observed in various cancer types	^18^F-FDG
Amino acids	Amino acid transporter is upregulated in carcinomas because of increased amino acid use for energy requirements and protein synthesis	^18^F-fluciclovine, ^11^C-methionine, ^11^C-serotonin, ^18^F-fluoro-methyl-arabinofuranosyl-uracil (FMAU)
PMSA	PSMA, a transmembrane cell protein, is heterogeneously expressed by normal prostate luminal epithelial cells and highly upregulated in PCa [15]	capromab pendetide (Prostascint), ^89^Zr-labeled J591^111^In-labeled J591, IAB2M,^68^Ga-PSMA-11, ^18^F-DCFPyL, ^99^mTc-MIP-1404
GRPR	GRPR is overexpressed in many cancer types, including PCa, stimulating tumor cell proliferation when activated by agonistic molecules, while being non-detectable in normal prostate tissues	^68^Ga-AMBA, ^68^Ga-BBN-RGD, ^111^In-RM1, ^68^Ga-RM2, ^68^Ga-SB3, NeoBOMB1, ^68^Ga-RM26
uPA ^1^	uPA binds with high affinity to uPAR, which is upregulated in most tumors, including PCa, and associated with advanced disease and poor prognosis [16]	^64^Cu-DOTA-AE105
PSCA ^2^	PSCA is overexpressed in 83–100% of PCas; the elevated level of PSCA expression is directly associated with advanced stages and high GSC	^89^Zr-A2cDb
STEAP1 ^3^	STEAP1 is overexpressed in many cancer cells, including PCa. STEAP1 shows a correlation between increased expression and tumor aggressiveness	^89^Zr-2109A
CD46	CD46 is overexpressed in primary tumor tissue and castration-resistant PCa	YS5 (^89^Zr-DFO-YS5)
AR	AR plays an important role in the growth of PCa because it is a transcription factor that influences many other genes, including the genes responsible for PSA	^18^F-Fluorodihydrotestosterone (^18^F-FDHT)
Bone seeking agents	A bone-seeking agent is physiologically absorbed at the site of osteoblastic activity and accumulates in sclerotic metastases, due to its high affinity to the newly formed bone	^18^F-NaF, ^99m^Tc-MDP

^1^ uPA—urokinase-type plasminogen activator; ^2^ PSCA—prostate stem cell antigen; ^3^ STEAP1—6 transmembrane epithelial antigen of prostate.

**Table 2:** Diagnostic performance of [^18^F]FACBC in the BCR of PCa.

Patients’ Condition	Imaging Modality	Detection Rate (%)	Reference
Number of patients: 213 GSC: ≥6 PSA (ng/mL): 4.24 (0.2–93.5) Mean age: 66.4 (46–90)	PET/CT	Overall: 57 31 (PSA: <0.5) 50 (PSA: 0.5–1.0) 66 (PSA: 1–2)	[40]
Number of patients: 84 GSC: ≥6 PSA (ng/mL): 0.7 (0.2–12.9) Mean age: 66.5 (53.0–84.7)	PET/MRI	Overall: 39.9 27.1 (PSA: <1) 55.6 (PSA: ≥1)	[38]
Number of patients: 328 GSC: unknown PSA (ng/mL): 1.6 (0.02–186.7) Mean age: 71(47–90)	PET/CT	Overall: 65 58 (PSA: 0–0.2) 50 (PSA: 0.2–0.5) 75 (PSA: 0.5–1.0) 73 (PSA: 1.0–2.0) 95 (PSA: 2.0–5.0) 97 (PSA: 5.0–10.0) 95 (PSA: ≥10.0)	[41]
Number of patients: 79 GSC: ≥6 PSA (ng/mL): 0.33 (0.02–31.00) Mean age: 61.6 ± 7.6	PET/CT	Overall: 79.7 75.4 (PSA: <1) 90.9 (PSA: ≥1)	[39]
Number of patients: 165 GSC: unknown PSA (ng/mL): 3.1 (0.02–2975) Mean age: 71.1 (51–91)	PET/CT	Overall: 66.7 15.4 (PSA: <0.5) 50 (PSA: 0.5–0.99) 55.6 (PSA: 1–2) 68.2 (PSA: 2–5) 93.7 (PSA: ≥5)	[37]
Number of patients: 81 GSC: unknown PSA (ng/mL): median 0.99 Mean age:74	PET/CT	Overall: 76.9 66.7 (PSA: 0.2–0.57) 71.4 (PSA: 0.58–0.99) 78.9 (PSA: 1–1.5) 90 (PSA: >1.5)	[43]

**Table 3:** The performance of [^68^Ga]Ga-PSMA-11 for BCR.

Patients’ Condition	Treatment	Imaging Modality	Detection Rate (%) (PSA:ng/mL)	Reference
Number of patients: 31 GSC: 7 (5–9) PSA (ng/mL): 2.0 (0.1–130.0) Mean age: 71 (51–78)	The combination or single use of RT, RP, HDR brachytherapy, and ADT and HIFU	dPET/CT and whole-body PET/CT	Overall: 71 46.7 (PSA: 0.1–2) 75.0 (PSA: >2)	[81]
Number of patients: 56 GSC: 6–9 (7/56 unknown) PSA (ng/mL): 0.99 (IQR: 3.1) Mean age: 69 (IQR: 11)	RP	PET/MRI	Overall: 78.6 44.4 (PSA: <0.2); 72.7 (PSA: 0.2–0.5) 80.0 (PSA: 0.5–2.0) 95.2 (PSA: >2.0)	[82]
Number of patients: 1007 GSC: 7 (5–10) PSA (ng/mL): 2.2 (0.01–1237) Mean age: 68 (39–90)	The combination or single use of RP, RT, ADT and HIFU	PET/CT	Overall: 79.5 46 (PSA: ≤0.2); 46 (PSA: 0.21–0.5) 73 (PSA: 0.51–1.0) 80 (PSA: 1.1–2.0) 86 (PSA: 2.1–3.0) 91 (PSA: 3.1–5.0) 94 (PSA: 5.1–7.0) 91 (PSA: 7.1–10.0) 96 (PSA: >10.0)	[76]
Number of patients: 270 GSC: ≥6 (6% unknown) PSA (ng/mL): 0.44 (0.03–1) Mean age: 68 (43–90)	RP without RT	PET/CT	Overall: 49	[83]
Number of patients: 119 GSC: ≥6 PSA (ng/mL): 0.32 (0.2–0.5) Mean age: 66 (44–78)	RP without RT after recurrence	PET/CT	Overall: 34.4	[84]
Number of patients: 223 PSA (ng/mL): 0.65 (0.2–8.9) Mean age: 70 (66–75)	RT, ADT-free six months before PET scan	PET/CT	Overall: 39.9 23.2 (PSA: 0.2–0.5) 49.6 (PSA: >0.5)	[78]
Number of patients: 660 GSC: >6, 45 patients GSC ≤ 6 PSA (ng/mL): median PSA 10.65 Mean age: 70 (49–88)	RP, RT, or brachytherapy	PET/CT	Overall: 76 41 (PSA: <0.2) 44.7 (PSA: 0.2–0.5) 61.7 (PSA: 0.5–1.0) 72.3 (PSA: 1.0–2.0) 82.5 (PSA: 2.0–5.0) 94 (PSA: ≥5)	[85]
Number of patients: 581 GSC: >6, 37 patients GSC ≤ 6 PSA (ng/mL): 2.98 (0.2–2000) Mean age: 71 (49–88)	RP, RT, and ADT	PET/CT	Overall: 77 Overall Post-RP: 75 40 (PSA: 0.2–0.5) 62 (PSA: 0.5–1) 70 (PSA: 1–2) 84 (PSA: 2–5) 94 (PSA: ≥5) Overall p-RT: 9188 (PSA: 2–5) 93 (PSA: ≥5)	[86]
Number of patients: 84 GSC: ≥8 PSA (ng/mL): 4.27 (0.8–18) Mean age: 71 (66–76)	Prior therapy: RT or RP Ongoing therapy: ADT	PET/CT or PET/MRI	Overall: 94 85.2 (PSA: 0–1) 97.3 (PSA: 1–20) 100 (PSA: >20)	[87]

**Table 4:** The diagnostic performance of [^11^C]choline, [^18^F]FACBC, [^68^Ga]Ga-PSMA-11, and [^18^F]DCFPyL from meta-analysis.

Imaging Agent	Pooled Sensitivity, %	Pooled Specificity, %	Pooled Detection Rate, %	References
[^11^C]choline	80–83	84–92	58–62.2	[108,109]
[^18^F]FACBC	79.7–88	61.9–73	58.6	[108,110]
[^68^Ga]Ga-PSMA-11	74–97	66–99.8	63–82.8	[73,108,111]
[^18^F]DCFPyL	91	90	81–92%	[93,112]

## Data Availability

Not applicable.

## Abbreviations

ADT: androgen deprivation therapy; AR: androgen receptor; BCR: biochemical recurrence; CRPC: castration-resistant prostate cancer; CT: computed tomography; ^18^F-FACBC: ^18^F-fluciclovine; FDA: Food and Drug Administration; GRPR: gastrin-releasing peptide receptor; GSC: Gleason score; MRI: magnetic resonance imaging; OS: overall survival; PCa: prostate cancer; PET: positron emission tomography; PSA: prostate-specific antigen; PSMA: prostate-specific membrane antigen; RP: radical prostatectomy; RT: radiotherapy; SPECT: single-photon emission computed tomography; SUVmax: sum of maximum standardized uptake value; TcBS: ^99m^Tc-MDP bone scan; TRUS: transrectal ultrasound.
